# Supplementary material for: Adenosine 2B Receptor Signaling Impairs Vaccine-Mediated Protection Against Pneumococcal Infection in Young Hosts by Blunting Neutrophil Killing of Antibody-Opsonized Bacteria
Source: Vaccines (Basel). 2025 Apr 15;13(4):414. doi: 10.3390/vaccines13040414 (PMC12031446; doi:10.3390/vaccines13040414)
Supplement: Supplementary file 1 [file vaccines-13-00414-s001.zip › vaccines-3564844-supplementary.pdf]

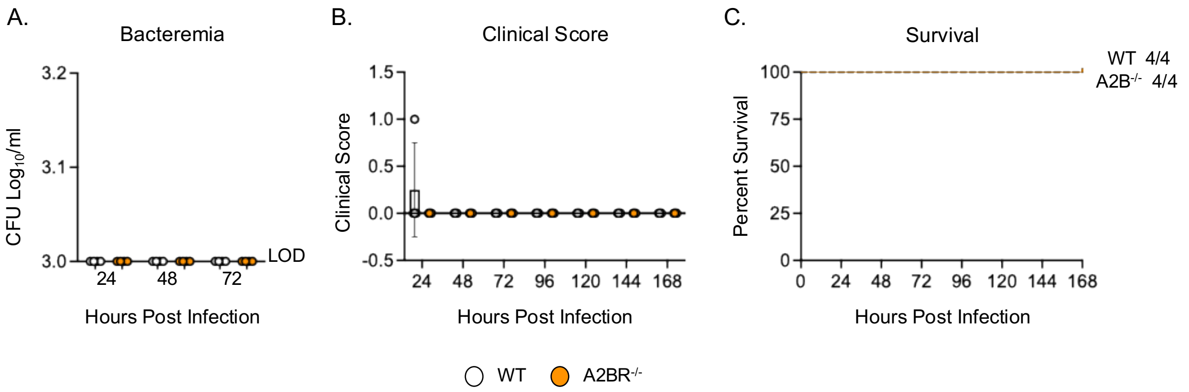


**Supplemental Figure S1: Vaccine efficacy in WT and A2BR$^{-/-}$ mice.** Young (2-3 months) WT C57BL/6J and A2BR$^{-/-}$ were vaccinated with PCV and 4 weeks later infected i.t with 2x10$^{6}$ CFU of *S. pneumoniae* TIGR4. At 25, 48, and 72 hpi blood was collected and plated for CFU to assess bacteremia (A). Mice were also monitored for 7 days and assessed for clinical signs of disease (B) and survival (C). Pooled data from n=4 mice per group are shown.
